# *catena*-Poly[[tetra-μ-benzoato-κ^8^ *O*:*O*′-dicopper(II)]-μ-[*N*-(pyridin-4-yl)nicotin­amide]-κ^2^ *N*:*N*′-[dibenzoato-κ^2^ *O*-copper(II)]-μ-[*N*-(pyridin-4-yl)nicotin­amide]-κ^2^ *N*:*N*′]

**DOI:** 10.1107/S1600536812030437

**Published:** 2012-07-10

**Authors:** Peter E. Kraft, Robert L. LaDuca

**Affiliations:** a Munster High School, Munster, IN 46321, USA; b Lyman Briggs College, Department of Chemistry, Michigan State University, East Lansing, MI 48825, USA

## Abstract

In the polymeric title compound, [Cu_3_(C_7_H_5_O_2_)_6_(C_11_H_9_N_3_O)_2_]_*n*_, square-planar-coordinated Cu^II^ ions on crystallographic inversion centres are bound by two monodentate benzoate anions. The resulting [Cu(benzoate)]_2_ fragments are joined to centrosymmetic [Cu_2_(benzoate)_4_] paddlewheel clusters [Cu⋯Cu = 2.6331 (5) Å] by means of bridging *N*-(pyridin-4-yl)nicotinamide (4-pna) ligands [dihedral angle between the aromatic rings = 39.18 (12)°], thereby forming [Cu_3_(benzoate)_6_(4-pna)_2_]_*n*_ coordination-polymer chains that are arranged parallel to the [30-1] crystal direction. These polymeric chains are anchored into supra­molecular layers by N—H⋯O hydrogen bonding between neighboring 4-pna ligands. These layers aggregate by crystal packing forces to afford the crystal structure of the title compound.

## Related literature

For the preparation of *N*-(pyridin-4-yl)­nicotinamide, see: Gardner *et al.* (1954 ▶). For the preparation of other coordination polymers containing *N*-(pyridin-4-yl)­nicotinamide, see: Kumar (2009 ▶).
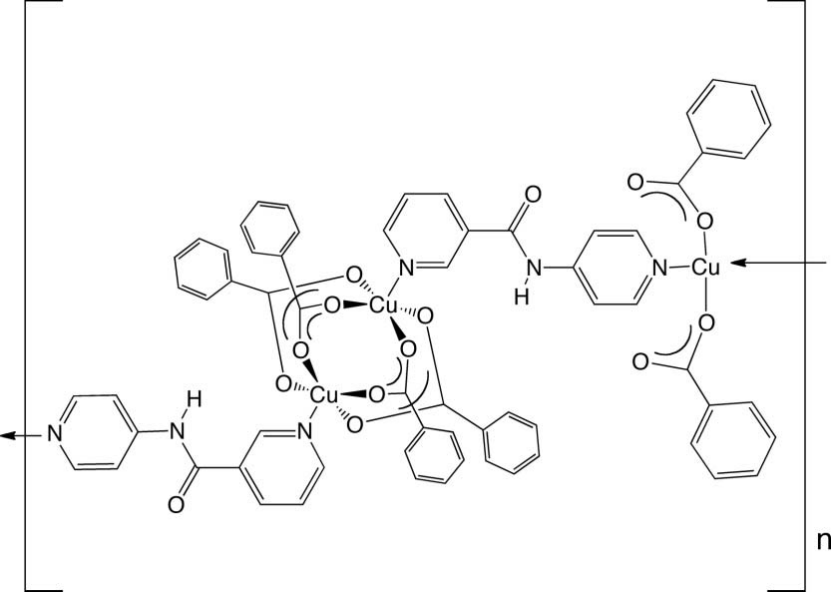


## Experimental

### 

#### Crystal data

[Cu_3_(C_7_H_5_O_2_)_6_(C_11_H_9_N_3_O)_2_]
*M* *_r_* = 1315.70
Triclinic, 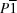

*a* = 8.9183 (7) Å
*b* = 11.3348 (9) Å
*c* = 15.5534 (12) Å
α = 85.471 (1)°
β = 75.804 (1)°
γ = 75.007 (1)°
*V* = 1472.1 (2) Å^3^
*Z* = 1
Mo *K*α radiation
μ = 1.15 mm^−1^
*T* = 173 K
0.30 × 0.20 × 0.20 mm

#### Data collection

Bruker APEXII CCD diffractometer
Absorption correction: multi-scan (*SADABS*; Sheldrick, 1996 ▶) *T*_min_ = 0.725, *T*_max_ = 0.803
24050 measured reflections
5372 independent reflections
4042 reflections with *I* > 2σ(*I*)
*R*_int_ = 0.053

#### Refinement

*R*[*F* ^2^ > 2σ(*F* ^2^)] = 0.035
*wR*(*F* ^2^) = 0.081
*S* = 1.02
5372 reflections
397 parameters
1 restraint
H atoms treated by a mixture of independent and constrained refinement
Δρ_max_ = 0.38 e Å^−3^
Δρ_min_ = −0.31 e Å^−3^

### 

Data collection: *APEX2* (Bruker, 2006 ▶); cell refinement: *SAINT* (Bruker, 2006 ▶); data reduction: *SAINT*; program(s) used to solve structure: *SHELXS97* (Sheldrick, 2008 ▶); program(s) used to refine structure: *SHELXL97* (Sheldrick, 2008 ▶); molecular graphics: *Crystal Maker* (Palmer, 2007 ▶); software used to prepare material for publication: *SHELXL97*.

## Supplementary Material

Crystal structure: contains datablock(s) I, global. DOI: 10.1107/S1600536812030437/hb6888sup1.cif

Structure factors: contains datablock(s) I. DOI: 10.1107/S1600536812030437/hb6888Isup2.hkl

Additional supplementary materials:  crystallographic information; 3D view; checkCIF report

## Figures and Tables

**Table 1:** Selected bond lengths (Å)

Cu1—O1	1.9481 (18)
Cu1—N1	2.014 (2)
Cu2—O4^i^	1.9543 (19)
Cu2—O6^i^	1.962 (2)
Cu2—O3	1.9727 (19)
Cu2—O5	1.973 (2)
Cu2—N3	2.196 (2)

Symmetry code: (i) 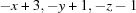.

**Table 2:** Hydrogen-bond geometry (Å, °)

*D*—H⋯*A*	*D*—H	H⋯*A*	*D*⋯*A*	*D*—H⋯*A*
N2—H2N⋯O2^ii^	0.86 (2)	2.01 (2)	2.867 (3)	177 (3)

Symmetry code: (ii) 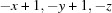.

**Fig. 1.**
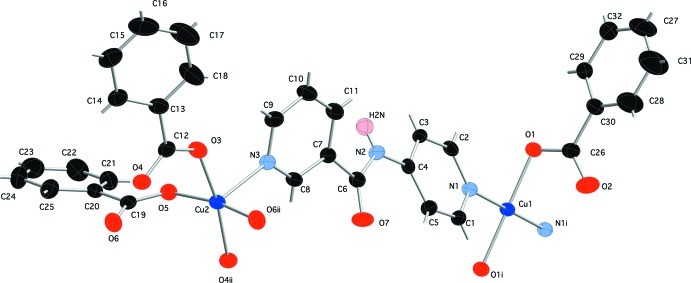
The asymmetric unit of the title compound, showing 50% probability ellipsoids, complete coordination environments, and atom numbering scheme. Hydrogen atom positions are shown as grey sticks with the exception of the amide group hydrogen atom. Color codes: dark blue Cu, red O, light blue N, black C, pink H. Symmetry codes: (i) -x, -y + 1, -z; (ii) -x + 3, -y + 1, -z - 1.

**Fig. 2.**
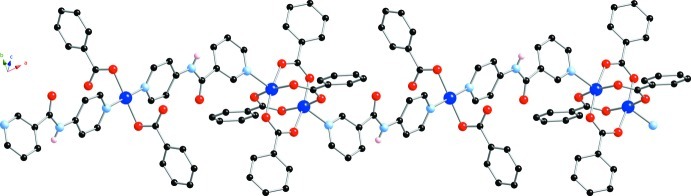
A single [Cu3(benzoate)6(4-pna)2]n coordination polymer chain.

**Fig. 3.**
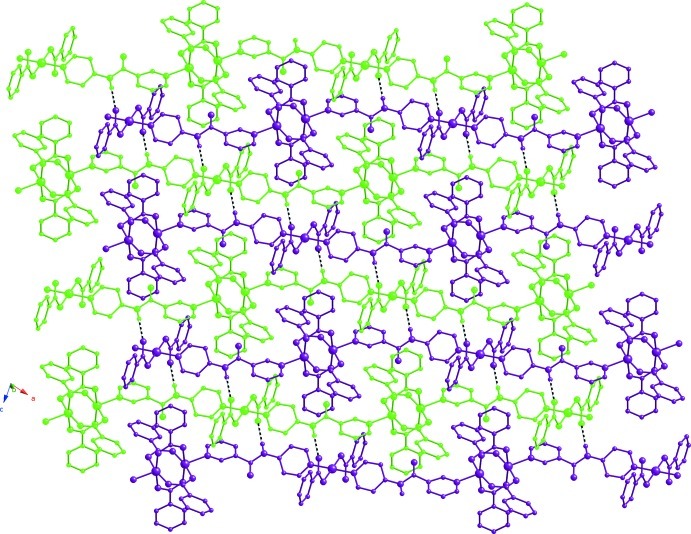
Supramolecular layer of [Cu3(benzoate)6(4-pna)2]n chains. N—H···O hydrogen bonding is shown as dashed lines.

**Fig. 4.**
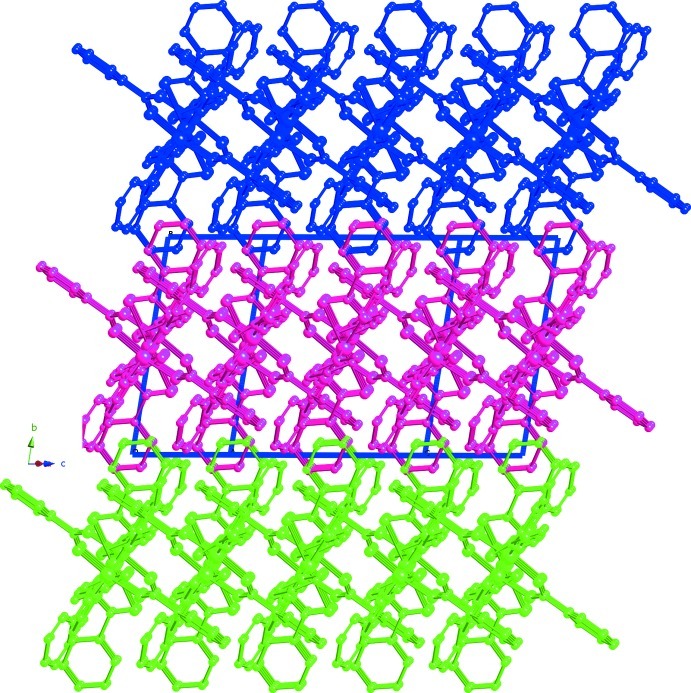
Stacking of supramolecular layers within the title compound.

## supplementary crystallographic information

### Comment

In comparison to divalent metal coordination polymers containing rigid rod dipyridine ligands such as 4,4'-bipyridine, related materials containing the kinked dipodal ligand *N*-(pyridin-4-yl)nicotinamide (4-pna) are much less common (Kumar, 2009). The title compound was obtained as blue crystals through the hydrothermal reaction of copper nitrate, benzoic acid, and 4-pna.

The asymmetric unit of the title compound (Fig. 1) contains two copper atoms, one of which (Cu1) lies on a crystallographic inversion centre, a 4-pna ligand, and three benzoate ligands. Cu1 is square planar coordinated by *trans* 4-pyridyl N atom donors from two 4-pna ligands and *trans* O atom donors from monodentate carboxylate groups belonging to two benzoate ligands. The other copper atom (Cu2) is square pyramidally coordinated, with its apical position occupied by a N atom donor from the nicotinamide end of a 4-pna ligand and its basal positions filled by O atom donors from four benzoate ligands.

Pairs of Cu2 atoms are linked into dinuclear [Cu_2_(benzoate)_4_] paddlewheel clusters by four *syn-syn* bridging benzoate ligands, with crystallographic inversion centres at the centroids of the clusters. Within these [Cu_2_(benzoate)_4_] clusters, the Cu···Cu through-space distance is 2.633 (2) Å. The 4-pna ligands projecting out of the apical positions of the Cu2 atoms within the dinuclear paddlewheel clusters connect to Cu1 atoms, generating one-dimensional [Cu_3_(benzoate)_6_(4-pna)_2_]_n_ polymer chains along the [3 0 1] crystal direction (Fig. 2).

Each individual chain is anchored to two others *via* N—H···O hydrogen bonding (Table 1) between amide moieties of neighboring 4-pna ligands. In this manner, supramolecular two-dimensional layers are constructed (Fig. 3); these lie parallel to the *ac* crystal planes. The three-dimensional structure of the title compound results from crystal packing forces between these layers, which stack along the *b* crystal direction. (Fig. 4).

### Experimental

Copper(II) nitrate hydrate and benzoic acid were obtained commercially. *N*-(Pyridin-4-yl)nicotinamide (4-pna) was prepared *via* a published procedure (Gardner *et al.*, 1954). A mixture of copper nitrate hydrate (89 mg, 0.37 mmol), benzoic acid (45 mg, 0.37 mmol), 4-pna (74 mg, 0.37 mmol) and 10.0 g water (550 mmol) was placed into a 23 ml Teflon-lined Parr acid digestion bomb, which was then heated under autogenous pressure at 393 K for 24 h. Blue blocks of the title compound were obtained.

### Refinement

All H atoms bound to C atoms were placed in calculated positions, with C—H = 0.95 Å, and refined in riding mode with *U*_iso_ = 1.2*U*_eq_(C). The H atom within the amide group of the 4-pna ligand was found in a difference Fourier map, restrained with N—H = 0.9 Å and refined with *U*_iso_ = 1.2*U*_eq_(N).

### Crystal data

**Table:**

[Cu_3_(C_7_H_5_O_2_)_6_(C_11_H_9_N_3_O)_2_]	*Z* = 1
*M**_r_* = 1315.70	*F*(000) = 673
Triclinic, *P*1	*D*_x_ = 1.484 Mg m^−^^3^
Hall symbol: -P 1	Mo *K*α radiation, λ = 0.71073 Å
*a* = 8.9183 (7) Å	Cell parameters from 24050 reflections
*b* = 11.3348 (9) Å	θ = 1.9–25.4°
*c* = 15.5534 (12) Å	µ = 1.15 mm^−^^1^
α = 85.471 (1)°	*T* = 173 K
β = 75.804 (1)°	Block, blue
γ = 75.007 (1)°	0.30 × 0.20 × 0.20 mm
*V* = 1472.1 (2) Å^3^	

### Data collection

**Table:**

Bruker APEXII CCD diffractometer	5372 independent reflections
Radiation source: fine-focus sealed tube	4042 reflections with *I* > 2σ(*I*)
Graphite monochromator	*R*_int_ = 0.053
ω–φ scans	θ_max_ = 25.4°, θ_min_ = 1.9°
Absorption correction: multi-scan (*SADABS*; Sheldrick, 1996)	*h* = −10→10
*T*_min_ = 0.725, *T*_max_ = 0.803	*k* = −13→13
24050 measured reflections	*l* = −18→18

### Refinement

**Table:**

Refinement on *F*^2^	Primary atom site location: structure-invariant direct methods
Least-squares matrix: full	Secondary atom site location: difference Fourier map
*R*[*F*^2^ > 2σ(*F*^2^)] = 0.035	Hydrogen site location: inferred from neighbouring sites
*wR*(*F*^2^) = 0.081	H atoms treated by a mixture of independent and constrained refinement
*S* = 1.02	*w* = 1/[σ^2^(*F*_o_^2^) + (0.0281*P*)^2^ + 1.0621*P*] where *P* = (*F*_o_^2^ + 2*F*_c_^2^)/3
5372 reflections	(Δ/σ)_max_ = 0.001
397 parameters	Δρ_max_ = 0.38 e Å^−^^3^
1 restraint	Δρ_min_ = −0.31 e Å^−^^3^

### Special details

**Table:**

Geometry. All e.s.d.'s (except the e.s.d. in the dihedral angle between two l.s. planes) are estimated using the full covariance matrix. The cell e.s.d.'s are taken into account individually in the estimation of e.s.d.'s in distances, angles and torsion angles; correlations between e.s.d.'s in cell parameters are only used when they are defined by crystal symmetry. An approximate (isotropic) treatment of cell e.s.d.'s is used for estimating e.s.d.'s involving l.s. planes.
Refinement. Refinement of *F*^2^ against ALL reflections. The weighted *R*-factor *wR* and goodness of fit *S* are based on *F*^2^, conventional *R*-factors *R* are based on *F*, with *F* set to zero for negative *F*^2^. The threshold expression of *F*^2^ > σ(*F*^2^) is used only for calculating *R*-factors(gt) *etc*. and is not relevant to the choice of reflections for refinement. *R*-factors based on *F*^2^ are statistically about twice as large as those based on *F*, and *R*- factors based on ALL data will be even larger.

### Fractional atomic coordinates and isotropic or equivalent isotropic displacement parameters (Å^2^)

**Table:**

	*x*	*y*	*z*	*U*_iso_*/*U*_eq_	
Cu1	0.0000	0.5000	0.0000	0.02146 (13)	
Cu2	1.36616 (4)	0.54604 (3)	−0.44207 (2)	0.02392 (11)	
O1	0.0790 (2)	0.35964 (17)	−0.07773 (12)	0.0266 (5)	
O2	0.2143 (3)	0.29187 (19)	0.02586 (14)	0.0394 (5)	
O3	1.4882 (2)	0.66396 (18)	−0.43039 (13)	0.0323 (5)	
O4	1.7183 (2)	0.58342 (18)	−0.52675 (13)	0.0295 (5)	
O5	1.4691 (2)	0.43007 (18)	−0.35983 (13)	0.0284 (5)	
O6	1.7006 (2)	0.35647 (18)	−0.45767 (13)	0.0329 (5)	
O7	0.7243 (2)	0.5482 (2)	−0.28967 (13)	0.0373 (5)	
N1	0.1981 (3)	0.5589 (2)	−0.05294 (14)	0.0218 (5)	
N2	0.6435 (3)	0.6367 (2)	−0.15343 (15)	0.0231 (5)	
H2N	0.684 (3)	0.661 (2)	−0.1152 (16)	0.028*	
N3	1.1596 (3)	0.6361 (2)	−0.33950 (14)	0.0227 (5)	
C1	0.2737 (3)	0.5403 (2)	−0.13860 (18)	0.0244 (6)	
H1	0.2240	0.5075	−0.1753	0.029*	
C2	0.2702 (3)	0.6069 (2)	−0.00283 (18)	0.0237 (6)	
H2	0.2170	0.6245	0.0573	0.028*	
C3	0.4166 (3)	0.6317 (2)	−0.03425 (18)	0.0226 (6)	
H3	0.4645	0.6631	0.0041	0.027*	
C4	0.4948 (3)	0.6105 (2)	−0.12310 (17)	0.0207 (6)	
C5	0.4182 (3)	0.5656 (2)	−0.17648 (18)	0.0238 (6)	
H5	0.4651	0.5529	−0.2379	0.029*	
C6	0.7466 (3)	0.6081 (3)	−0.23565 (18)	0.0248 (6)	
C7	0.8914 (3)	0.6583 (3)	−0.25392 (17)	0.0222 (6)	
C8	1.0264 (3)	0.5974 (3)	−0.31442 (18)	0.0239 (6)	
H8	1.0235	0.5243	−0.3392	0.029*	
C9	1.1613 (3)	0.7396 (3)	−0.30381 (18)	0.0298 (7)	
H9	1.2558	0.7681	−0.3207	0.036*	
C10	1.0331 (3)	0.8063 (3)	−0.2440 (2)	0.0327 (7)	
H10	1.0392	0.8791	−0.2202	0.039*	
C11	0.8949 (3)	0.7656 (3)	−0.21908 (19)	0.0283 (7)	
H11	0.8038	0.8107	−0.1786	0.034*	
C12	1.6289 (3)	0.6623 (3)	−0.47145 (19)	0.0270 (7)	
C13	1.6959 (4)	0.7642 (3)	−0.45442 (19)	0.0306 (7)	
C14	1.8593 (4)	0.7500 (3)	−0.47238 (19)	0.0313 (7)	
H14	1.9295	0.6754	−0.4957	0.038*	
C15	1.9208 (4)	0.8441 (3)	−0.4565 (2)	0.0396 (8)	
H15	2.0332	0.8336	−0.4688	0.047*	
C16	1.8214 (5)	0.9517 (3)	−0.4232 (2)	0.0566 (11)	
H16	1.8646	1.0162	−0.4130	0.068*	
C17	1.6585 (5)	0.9670 (4)	−0.4045 (3)	0.0756 (15)	
H17	1.5888	1.0419	−0.3812	0.091*	
C18	1.5972 (4)	0.8726 (3)	−0.4199 (3)	0.0623 (12)	
H18	1.4848	0.8829	−0.4065	0.075*	
C19	1.6096 (3)	0.3635 (3)	−0.38105 (19)	0.0271 (7)	
C20	1.6740 (3)	0.2846 (3)	−0.31014 (19)	0.0275 (7)	
C21	1.5723 (4)	0.2589 (3)	−0.2323 (2)	0.0342 (7)	
H21	1.4603	0.2916	−0.2235	0.041*	
C22	1.6334 (4)	0.1856 (3)	−0.1674 (2)	0.0453 (9)	
H22	1.5631	0.1666	−0.1147	0.054*	
C23	1.7952 (5)	0.1399 (3)	−0.1787 (2)	0.0519 (10)	
H23	1.8370	0.0905	−0.1337	0.062*	
C24	1.8963 (4)	0.1663 (3)	−0.2558 (3)	0.0511 (10)	
H24	2.0084	0.1350	−0.2639	0.061*	
C25	1.8364 (4)	0.2375 (3)	−0.3211 (2)	0.0402 (8)	
H25	1.9073	0.2544	−0.3743	0.048*	
C26	0.1744 (3)	0.2772 (3)	−0.04270 (19)	0.0263 (7)	
C27	0.3730 (5)	−0.0630 (4)	−0.1756 (3)	0.0698 (13)	
H27	0.4193	−0.1385	−0.2053	0.084*	
C28	0.3120 (5)	0.0550 (3)	−0.0454 (3)	0.0555 (10)	
H28	0.3169	0.0601	0.0145	0.067*	
C29	0.2334 (4)	0.1471 (3)	−0.1756 (2)	0.0368 (8)	
H29	0.1829	0.2163	−0.2058	0.044*	
C30	0.2388 (4)	0.1566 (3)	−0.0885 (2)	0.0326 (7)	
C31	0.3782 (6)	−0.0545 (4)	−0.0896 (3)	0.0787 (14)	
H31	0.4278	−0.1244	−0.0596	0.094*	
C32	0.3013 (4)	0.0368 (3)	−0.2192 (3)	0.0514 (10)	
H32	0.2980	0.0309	−0.2793	0.062*	

### Atomic displacement parameters (Å^2^)

**Table:**

	*U*^11^	*U*^22^	*U*^33^	*U*^12^	*U*^13^	*U*^23^
Cu1	0.0145 (2)	0.0247 (3)	0.0243 (3)	−0.0074 (2)	0.0022 (2)	−0.0077 (2)
Cu2	0.01624 (19)	0.0281 (2)	0.0262 (2)	−0.00850 (15)	0.00295 (14)	−0.00849 (15)
O1	0.0183 (10)	0.0285 (11)	0.0325 (11)	−0.0079 (9)	0.0004 (9)	−0.0107 (9)
O2	0.0537 (15)	0.0391 (13)	0.0320 (12)	−0.0183 (11)	−0.0145 (11)	−0.0026 (10)
O3	0.0212 (11)	0.0328 (12)	0.0422 (13)	−0.0136 (9)	0.0051 (9)	−0.0123 (10)
O4	0.0211 (11)	0.0349 (12)	0.0336 (12)	−0.0133 (9)	0.0016 (9)	−0.0109 (10)
O5	0.0205 (11)	0.0332 (12)	0.0298 (11)	−0.0065 (9)	−0.0002 (9)	−0.0082 (9)
O6	0.0234 (11)	0.0386 (13)	0.0314 (12)	−0.0043 (9)	0.0019 (9)	−0.0065 (10)
O7	0.0288 (12)	0.0510 (14)	0.0341 (12)	−0.0216 (11)	0.0079 (9)	−0.0220 (11)
N1	0.0179 (12)	0.0237 (13)	0.0232 (12)	−0.0074 (10)	−0.0001 (10)	−0.0051 (10)
N2	0.0163 (12)	0.0310 (14)	0.0224 (13)	−0.0101 (10)	0.0012 (10)	−0.0068 (11)
N3	0.0182 (12)	0.0272 (13)	0.0220 (12)	−0.0088 (10)	0.0008 (10)	−0.0036 (10)
C1	0.0191 (15)	0.0285 (16)	0.0260 (16)	−0.0066 (12)	−0.0036 (12)	−0.0060 (12)
C2	0.0206 (15)	0.0227 (15)	0.0241 (15)	−0.0041 (12)	0.0020 (12)	−0.0056 (12)
C3	0.0176 (14)	0.0257 (16)	0.0262 (15)	−0.0086 (12)	−0.0028 (12)	−0.0069 (12)
C4	0.0159 (14)	0.0195 (14)	0.0250 (15)	−0.0045 (11)	−0.0012 (12)	−0.0013 (12)
C5	0.0203 (15)	0.0299 (16)	0.0201 (15)	−0.0074 (13)	−0.0006 (12)	−0.0037 (12)
C6	0.0205 (15)	0.0260 (16)	0.0271 (16)	−0.0085 (12)	−0.0001 (12)	−0.0034 (13)
C7	0.0176 (14)	0.0267 (15)	0.0207 (14)	−0.0084 (12)	0.0021 (11)	−0.0024 (12)
C8	0.0211 (15)	0.0246 (15)	0.0247 (15)	−0.0073 (12)	0.0004 (12)	−0.0059 (12)
C9	0.0242 (16)	0.0379 (18)	0.0288 (16)	−0.0156 (14)	0.0015 (13)	−0.0071 (14)
C10	0.0305 (17)	0.0300 (17)	0.0364 (18)	−0.0144 (14)	0.0053 (14)	−0.0122 (14)
C11	0.0230 (16)	0.0306 (17)	0.0271 (16)	−0.0066 (13)	0.0043 (13)	−0.0075 (13)
C12	0.0228 (16)	0.0312 (17)	0.0297 (16)	−0.0137 (13)	−0.0033 (13)	−0.0030 (14)
C13	0.0301 (17)	0.0326 (18)	0.0309 (17)	−0.0170 (14)	0.0007 (13)	−0.0061 (14)
C14	0.0293 (17)	0.0373 (18)	0.0308 (17)	−0.0174 (14)	−0.0030 (13)	−0.0026 (14)
C15	0.043 (2)	0.052 (2)	0.0340 (18)	−0.0301 (18)	−0.0080 (15)	0.0037 (16)
C16	0.068 (3)	0.052 (2)	0.058 (2)	−0.043 (2)	0.003 (2)	−0.014 (2)
C17	0.060 (3)	0.045 (2)	0.116 (4)	−0.026 (2)	0.018 (3)	−0.046 (2)
C18	0.036 (2)	0.045 (2)	0.100 (3)	−0.0187 (18)	0.015 (2)	−0.032 (2)
C19	0.0240 (16)	0.0270 (16)	0.0336 (17)	−0.0112 (13)	−0.0044 (14)	−0.0105 (13)
C20	0.0265 (16)	0.0263 (16)	0.0316 (17)	−0.0066 (13)	−0.0076 (13)	−0.0083 (13)
C21	0.0302 (18)	0.0311 (18)	0.0398 (19)	−0.0069 (14)	−0.0057 (15)	−0.0022 (15)
C22	0.049 (2)	0.043 (2)	0.039 (2)	−0.0082 (18)	−0.0059 (17)	0.0021 (17)
C23	0.069 (3)	0.045 (2)	0.044 (2)	−0.003 (2)	−0.028 (2)	−0.0031 (17)
C24	0.035 (2)	0.061 (3)	0.059 (2)	−0.0002 (18)	−0.0238 (19)	−0.011 (2)
C25	0.0311 (19)	0.051 (2)	0.041 (2)	−0.0112 (16)	−0.0095 (15)	−0.0088 (17)
C26	0.0228 (16)	0.0294 (17)	0.0273 (16)	−0.0131 (13)	0.0015 (13)	−0.0055 (13)
C27	0.083 (3)	0.037 (2)	0.087 (3)	0.006 (2)	−0.030 (3)	−0.030 (2)
C28	0.080 (3)	0.034 (2)	0.052 (2)	−0.008 (2)	−0.021 (2)	−0.0035 (18)
C29	0.0276 (17)	0.0356 (19)	0.048 (2)	−0.0015 (14)	−0.0128 (15)	−0.0141 (16)
C30	0.0291 (17)	0.0278 (17)	0.0411 (19)	−0.0067 (14)	−0.0059 (14)	−0.0100 (14)
C31	0.118 (4)	0.029 (2)	0.088 (3)	0.003 (2)	−0.044 (3)	−0.004 (2)
C32	0.047 (2)	0.048 (2)	0.060 (2)	0.0019 (18)	−0.0214 (19)	−0.0288 (19)

### Geometric parameters (Å, º)

**Table:**

Cu1—O1	1.9481 (18)	C10—C11	1.384 (4)
Cu1—O1^i^	1.9481 (18)	C10—H10	0.9500
Cu1—N1^i^	2.014 (2)	C11—H11	0.9500
Cu1—N1	2.014 (2)	C12—C13	1.497 (4)
Cu2—O4^ii^	1.9543 (19)	C13—C18	1.374 (4)
Cu2—O6^ii^	1.962 (2)	C13—C14	1.384 (4)
Cu2—O3	1.9727 (19)	C14—C15	1.382 (4)
Cu2—O5	1.973 (2)	C14—H14	0.9500
Cu2—N3	2.196 (2)	C15—C16	1.362 (5)
O1—C26	1.276 (3)	C15—H15	0.9500
O2—C26	1.239 (3)	C16—C17	1.377 (5)
O3—C12	1.257 (3)	C16—H16	0.9500
O4—C12	1.262 (3)	C17—C18	1.382 (5)
O4—Cu2^ii^	1.9543 (18)	C17—H17	0.9500
O5—C19	1.263 (3)	C18—H18	0.9500
O6—C19	1.262 (3)	C19—C20	1.495 (4)
O6—Cu2^ii^	1.962 (2)	C20—C25	1.381 (4)
O7—C6	1.203 (3)	C20—C21	1.384 (4)
N1—C1	1.343 (3)	C21—C22	1.382 (4)
N1—C2	1.346 (3)	C21—H21	0.9500
N2—C6	1.386 (3)	C22—C23	1.372 (5)
N2—C4	1.394 (3)	C22—H22	0.9500
N2—H2N	0.861 (17)	C23—C24	1.376 (5)
N3—C8	1.331 (3)	C23—H23	0.9500
N3—C9	1.341 (3)	C24—C25	1.371 (5)
C1—C5	1.373 (4)	C24—H24	0.9500
C1—H1	0.9500	C25—H25	0.9500
C2—C3	1.370 (4)	C26—C30	1.501 (4)
C2—H2	0.9500	C27—C31	1.362 (6)
C3—C4	1.395 (4)	C27—C32	1.366 (5)
C3—H3	0.9500	C27—H27	0.9500
C4—C5	1.392 (4)	C28—C30	1.381 (5)
C5—H5	0.9500	C28—C31	1.385 (5)
C6—C7	1.499 (4)	C28—H28	0.9500
C7—C11	1.380 (4)	C29—C30	1.380 (4)
C7—C8	1.389 (4)	C29—C32	1.389 (4)
C8—H8	0.9500	C29—H29	0.9500
C9—C10	1.374 (4)	C31—H31	0.9500
C9—H9	0.9500	C32—H32	0.9500
			
O1—Cu1—O1^i^	180.0	O3—C12—O4	125.9 (3)
O1—Cu1—N1^i^	89.20 (8)	O3—C12—C13	117.2 (3)
O1^i^—Cu1—N1^i^	90.80 (8)	O4—C12—C13	116.9 (2)
O1—Cu1—N1	90.80 (8)	C18—C13—C14	118.7 (3)
O1^i^—Cu1—N1	89.20 (8)	C18—C13—C12	120.9 (3)
N1^i^—Cu1—N1	180.0	C14—C13—C12	120.4 (3)
O4^ii^—Cu2—O6^ii^	88.63 (8)	C15—C14—C13	120.2 (3)
O4^ii^—Cu2—O3	168.50 (8)	C15—C14—H14	119.9
O6^ii^—Cu2—O3	89.13 (9)	C13—C14—H14	119.9
O4^ii^—Cu2—O5	88.78 (8)	C16—C15—C14	120.4 (3)
O6^ii^—Cu2—O5	168.47 (8)	C16—C15—H15	119.8
O3—Cu2—O5	91.18 (8)	C14—C15—H15	119.8
O4^ii^—Cu2—N3	99.17 (8)	C15—C16—C17	120.1 (3)
O6^ii^—Cu2—N3	96.15 (8)	C15—C16—H16	120.0
O3—Cu2—N3	92.29 (8)	C17—C16—H16	120.0
O5—Cu2—N3	95.35 (8)	C16—C17—C18	119.4 (4)
C26—O1—Cu1	108.01 (17)	C16—C17—H17	120.3
C12—O3—Cu2	126.41 (18)	C18—C17—H17	120.3
C12—O4—Cu2^ii^	119.08 (17)	C13—C18—C17	121.1 (3)
C19—O5—Cu2	123.94 (19)	C13—C18—H18	119.4
C19—O6—Cu2^ii^	121.68 (19)	C17—C18—H18	119.4
C1—N1—C2	116.7 (2)	O6—C19—O5	125.6 (3)
C1—N1—Cu1	121.09 (18)	O6—C19—C20	116.7 (3)
C2—N1—Cu1	121.88 (18)	O5—C19—C20	117.7 (3)
C6—N2—C4	126.4 (2)	C25—C20—C21	119.0 (3)
C6—N2—H2N	115.0 (19)	C25—C20—C19	120.3 (3)
C4—N2—H2N	117.6 (19)	C21—C20—C19	120.7 (3)
C8—N3—C9	117.4 (2)	C22—C21—C20	120.1 (3)
C8—N3—Cu2	123.03 (18)	C22—C21—H21	120.0
C9—N3—Cu2	119.43 (18)	C20—C21—H21	120.0
N1—C1—C5	124.1 (3)	C23—C22—C21	120.4 (3)
N1—C1—H1	117.9	C23—C22—H22	119.8
C5—C1—H1	117.9	C21—C22—H22	119.8
N1—C2—C3	123.3 (3)	C22—C23—C24	119.5 (3)
N1—C2—H2	118.4	C22—C23—H23	120.2
C3—C2—H2	118.4	C24—C23—H23	120.2
C2—C3—C4	119.4 (2)	C25—C24—C23	120.4 (3)
C2—C3—H3	120.3	C25—C24—H24	119.8
C4—C3—H3	120.3	C23—C24—H24	119.8
C5—C4—N2	123.9 (2)	C24—C25—C20	120.6 (3)
C5—C4—C3	117.8 (2)	C24—C25—H25	119.7
N2—C4—C3	118.3 (2)	C20—C25—H25	119.7
C1—C5—C4	118.7 (3)	O2—C26—O1	123.7 (3)
C1—C5—H5	120.7	O2—C26—C30	119.6 (3)
C4—C5—H5	120.7	O1—C26—C30	116.7 (3)
O7—C6—N2	123.9 (3)	C31—C27—C32	120.1 (4)
O7—C6—C7	121.2 (2)	C31—C27—H27	120.0
N2—C6—C7	114.9 (2)	C32—C27—H27	120.0
C11—C7—C8	118.2 (2)	C30—C28—C31	119.8 (4)
C11—C7—C6	124.0 (2)	C30—C28—H28	120.1
C8—C7—C6	117.7 (2)	C31—C28—H28	120.1
N3—C8—C7	123.4 (3)	C30—C29—C32	120.3 (3)
N3—C8—H8	118.3	C30—C29—H29	119.8
C7—C8—H8	118.3	C32—C29—H29	119.8
N3—C9—C10	123.2 (3)	C29—C30—C28	119.1 (3)
N3—C9—H9	118.4	C29—C30—C26	120.6 (3)
C10—C9—H9	118.4	C28—C30—C26	120.2 (3)
C9—C10—C11	118.8 (3)	C27—C31—C28	120.7 (4)
C9—C10—H10	120.6	C27—C31—H31	119.6
C11—C10—H10	120.6	C28—C31—H31	119.6
C7—C11—C10	118.9 (3)	C27—C32—C29	119.9 (3)
C7—C11—H11	120.5	C27—C32—H32	120.1
C10—C11—H11	120.5	C29—C32—H32	120.1
			
N1^i^—Cu1—O1—C26	−97.42 (18)	C6—C7—C11—C10	−176.8 (3)
N1—Cu1—O1—C26	82.58 (18)	C9—C10—C11—C7	1.1 (5)
O4^ii^—Cu2—O3—C12	−7.5 (6)	Cu2—O3—C12—O4	−1.2 (4)
O6^ii^—Cu2—O3—C12	−86.3 (2)	Cu2—O3—C12—C13	177.83 (19)
O5—Cu2—O3—C12	82.2 (2)	Cu2^ii^—O4—C12—O3	2.9 (4)
N3—Cu2—O3—C12	177.6 (2)	Cu2^ii^—O4—C12—C13	−176.08 (19)
O4^ii^—Cu2—O5—C19	91.6 (2)	O3—C12—C13—C18	−20.6 (5)
O6^ii^—Cu2—O5—C19	14.5 (5)	O4—C12—C13—C18	158.5 (3)
O3—Cu2—O5—C19	−76.9 (2)	O3—C12—C13—C14	158.7 (3)
N3—Cu2—O5—C19	−169.4 (2)	O4—C12—C13—C14	−22.2 (4)
O1—Cu1—N1—C1	32.9 (2)	C18—C13—C14—C15	−0.6 (5)
O1^i^—Cu1—N1—C1	−147.1 (2)	C12—C13—C14—C15	−179.9 (3)
O1—Cu1—N1—C2	−140.1 (2)	C13—C14—C15—C16	−0.2 (5)
O1^i^—Cu1—N1—C2	39.9 (2)	C14—C15—C16—C17	0.6 (6)
O4^ii^—Cu2—N3—C8	0.9 (2)	C15—C16—C17—C18	−0.2 (7)
O6^ii^—Cu2—N3—C8	90.5 (2)	C14—C13—C18—C17	1.0 (6)
O3—Cu2—N3—C8	179.9 (2)	C12—C13—C18—C17	−179.7 (4)
O5—Cu2—N3—C8	−88.7 (2)	C16—C17—C18—C13	−0.6 (7)
O4^ii^—Cu2—N3—C9	−174.6 (2)	Cu2^ii^—O6—C19—O5	0.8 (4)
O6^ii^—Cu2—N3—C9	−85.0 (2)	Cu2^ii^—O6—C19—C20	179.98 (17)
O3—Cu2—N3—C9	4.4 (2)	Cu2—O5—C19—O6	−3.9 (4)
O5—Cu2—N3—C9	95.8 (2)	Cu2—O5—C19—C20	176.99 (17)
C2—N1—C1—C5	0.8 (4)	O6—C19—C20—C25	19.3 (4)
Cu1—N1—C1—C5	−172.5 (2)	O5—C19—C20—C25	−161.5 (3)
C1—N1—C2—C3	−2.8 (4)	O6—C19—C20—C21	−161.4 (3)
Cu1—N1—C2—C3	170.4 (2)	O5—C19—C20—C21	17.8 (4)
N1—C2—C3—C4	2.1 (4)	C25—C20—C21—C22	−0.8 (4)
C6—N2—C4—C5	−9.2 (4)	C19—C20—C21—C22	179.8 (3)
C6—N2—C4—C3	171.6 (3)	C20—C21—C22—C23	1.4 (5)
C2—C3—C4—C5	0.6 (4)	C21—C22—C23—C24	−0.9 (5)
C2—C3—C4—N2	179.9 (2)	C22—C23—C24—C25	−0.1 (5)
N1—C1—C5—C4	1.8 (4)	C23—C24—C25—C20	0.6 (5)
N2—C4—C5—C1	178.3 (3)	C21—C20—C25—C24	−0.1 (5)
C3—C4—C5—C1	−2.5 (4)	C19—C20—C25—C24	179.2 (3)
C4—N2—C6—O7	−5.6 (5)	Cu1—O1—C26—O2	−4.5 (3)
C4—N2—C6—C7	174.1 (2)	Cu1—O1—C26—C30	175.13 (19)
O7—C6—C7—C11	149.7 (3)	C32—C29—C30—C28	−0.3 (5)
N2—C6—C7—C11	−30.0 (4)	C32—C29—C30—C26	176.3 (3)
O7—C6—C7—C8	−25.7 (4)	C31—C28—C30—C29	−0.1 (6)
N2—C6—C7—C8	154.6 (3)	C31—C28—C30—C26	−176.8 (4)
C9—N3—C8—C7	0.0 (4)	O2—C26—C30—C29	−162.1 (3)
Cu2—N3—C8—C7	−175.6 (2)	O1—C26—C30—C29	18.2 (4)
C11—C7—C8—N3	1.0 (4)	O2—C26—C30—C28	14.5 (4)
C6—C7—C8—N3	176.6 (3)	O1—C26—C30—C28	−165.1 (3)
C8—N3—C9—C10	−0.5 (4)	C32—C27—C31—C28	−0.3 (8)
Cu2—N3—C9—C10	175.3 (2)	C30—C28—C31—C27	0.4 (7)
N3—C9—C10—C11	−0.1 (5)	C31—C27—C32—C29	−0.1 (7)
C8—C7—C11—C10	−1.5 (4)	C30—C29—C32—C27	0.5 (5)

Symmetry codes: (i) −*x*, −*y*+1, −*z*; (ii) −*x*+3, −*y*+1, −*z*−1.

### Hydrogen-bond geometry (Å, º)

**Table:**

*D*—H···*A*	*D*—H	H···*A*	*D*···*A*	*D*—H···*A*
N2—H2*N*···O2^iii^	0.86 (2)	2.01 (2)	2.867 (3)	177 (3)

Symmetry code: (iii) −*x*+1, −*y*+1, −*z*.
